# Scientific and technological innovation cooperation network of the Greater Bay Area in South China: A social network analysis

**DOI:** 10.1371/journal.pone.0326515

**Published:** 2025-07-01

**Authors:** Chuyi Shen, Zhenjie Yang, Fat-iam Lam, Sio wa Tam, Wenping Yang, Yunxin Li

**Affiliations:** 1 Macao Polytechnic University, Macau, China; 2 Zhongnan University of Economics and Law, Wuhan, China; Guangzhou Institute of Geography, Guangdong Academy of Sciences, CHINA

## Abstract

Regional innovation cooperation focused upon either regions within one nation or transnational regions. Different from the discussion in the existing literature, the Guangdong- Hong Kong-Macao Greater Bay Area exists as an exceptional cross-border city-to-city cooperation under “one country, two systems”. Based on the social network analysis on co- patents in the Greater Bay Area, this paper aims to investigate the models and features of the innovation cooperation network of science and technology in the Greater Bay Area and the different characteristics of collaboration policy on cross-border knowledge flow before and after 2015. The results show that the innovation network of science and technology in the Greater Bay Area has a loosely structured pattern, with Hong Kong-Shenzhen-Guangzhou as a prominent hub. This structure portrays multiple centers that radiate out to its peripheries. The national policy launched in 2015 reduces the cooperation barriers and promotes the cross-border collaboration. The universities and research institutes possess significant intermediary roles and innovation autonomy in the scientific and technological innovation cooperation network of the Greater Bay Area.

## Introduction

Regional innovation cooperation is a vital strategy for driving scientific innovation and development as the generation of innovation is crucial for achieving regional growth objectives. Two literature streams have examined regional cooperation: one is focusing on cooperation within one nation, the other one is the cross-border collaboration between multiple countries within the region. The influencing factors and concerns varied between two different contexts. The conducive institutional environment, sufficient human resources, and the methodical innovation system have jointly contributed to promoting collaborative innovation across the region of one nation [1]. Without the border constraint, domestic cooperation fosters smoother knowledge flows when technological proximity is regulated [2]. However, cross-border regions encounter the challenge of connecting with nearby but inaccessible resources for knowledge and innovation due to the existence of borders [3]. Thus, scholars focused upon what factors contributed to the cross-border cooperation. Flows of information, trust, and technological competence were identified as crucial factors in cross-border cooperation [4]. Geographical, social, and cognitive similarities have been identified as key factors in shaping regional cooperation networks [5,6]. Universities and non-profit organizations could aid in creating environments of innovation, promoting cross-border knowledge exchange and cooperation in innovation [7]. The growth of trade, commerce, scientific and technological collaboration, and knowledge exchange in recent years has underscored the advantages of regional cooperation across borders [8,9]. The percentage of patents with a co-inventor from another nation has risen from 10% to 20% over the last three decades [10].

While borders were generally considered as the impediment to the regional cooperation, scholars observed that the borders have the positive role for the regional cooperation [11,12]. As borders are dynamic institutions, differences in culture and institutions can spur innovation. Scholars have suggested “bringing borders back into cross- border regional innovation cooperation” [13], emphasising the importance of border regions in promoting regional innovation. Based on patent data, related transaction data, and company data, one study reveals a significant inverted U-shaped correlation between financing constraints and innovation performance in the cross- border innovation cooperation network of internet enterprises [14]. It is important to note that financing constraints can affect innovation performance positively or negatively, depending on its level.

The literature on cross-border regional innovation to date has primarily centred on European Union (EU) regions [7]. In contrast to the regional partnership of the European Union and the San Francisco Bay Area, the Guangdong-Hong Kong-Macao Greater Bay Area stands out as an exceptional cross-border city-to-city cooperation within the framework of “one country, two systems” [15]. Guangdong, Hong Kong, and Macao operate within the same country and adhere to two systems and three types of laws. This cross-border cooperation provides an opportunity to study regional innovation systems in a unique institutional setting. In 2015, the national government promoted a policy to support scientific and technological innovation collaboration within the Greater Bay Area. *The Outline Development Plan for the Guangdong-Hong Kong-Macao Greater Bay Area*, issued by the Chinese Government, explicitly states the need to “build an open science, technology and innovation community, create high-level science, technology and innovation carriers and platforms, and optimize the environment for science, technology and innovation” [16]. This has set the tone for the cooperation and development of scientific and technological innovation in the Greater Bay Area. Following the implementation of this major initiative, the Greater Bay Area has witnessed strengthened interregional linkages and rapid economic development, with its annual economic growth rate reaching 5.93% from 2015 [17]. The coordinated urbanization development has been significantly enhanced, accompanied by intensified industrial clusters in high-tech industries and advanced manufacturing sectors. In the field of scientific innovation, the region benefits from an abundant talent pool and substantial R&D investments, fostering collaborative clusters among prestigious institutions such as The University of Hong Kong, Sun Yat-sen University, and University of Macao, as well as research organizations and enterprises of various scales. Compared to the previous year, the region witnessed an approximate 50% increase in invention patent applications in 2015. Specifically targeting the field of scientific and technological patents, the policy’s introduction of a pilot program for cross-border intellectual property pledging has significantly enhanced patent cooperation and financial support between Shenzhen and Hong Kong. Additionally, the policy grants joint R&D centers the same treatment as domestic R&D institutions, thereby fostering the improvement of applied basic research capabilities across the Greater Bay Area. This initiative not only strengthens collaboration in innovation but also provides a robust foundation for advancing the region’s scientific and technological prowess. [18,19].

Scientific and technological innovation in the Greater Bay Area has also attracted attention in the academia. In cross-regional innovation activities, technological and scientific knowledge are the main forms of innovation knowledge flow [20,21]. Collaborative research on scientific papers and technology transfer through collaborative patents lead to the spontaneous involvement of innovation subjects within the region in the innovation network, where they are influenced [22,23]. To clarify the forms of innovation networks and innovation activities, research focus on the performance of participants during innovation activities, analyzing the innovation capacity of subjects within the region and the impact of the overall network on participants’ innovation activities [24,25]. As research progresses, analyzing the diffusion of innovation knowledge from a spatial perspective provides new research insights for cross-border innovation activities. As an objective metric for assessing technological innovation, patent data provides quantifiable statistical indicators in empirical studies of regional innovation and intellectual property development. Compared to other indicators predominantly focused on academic domains, patent data more directly reflects the practical application of scientific and technological achievements [26]. Cai et al. examined the spatial pattern of technology transfer in the Greater Bay Area in terms of network nodes and hierarchy by using data on patented technology transactions [27]. Yang et al. focused on unraveling the intricate web of influences that steer patenting and transformation within 34 universities in the Greater Bay Area through examining university invention patents and transformation. Incorporating the unique “one country, two systems” policy of the Greater Bay Area as a variable, analyzing the spillover effects of patent transformation within the region and the factors influencing universities’ ability to enhance patent transformation [15].

The social network analysis is one of main research methods in these studies, and its application has enabled the academic community to have a deep understanding of the regional innovation system. Based on the behaviors of patent cooperation, collaborative thesis, and patent transfer, the use of network characteristics can be used to analyze the industrial clusters in multiple fields [28–30]. Among them, the patent cooperation network is one of the most objective and concise forms to reflect the inter-regional science and technology innovation linkage [31]. As one of the indicators of technological and intellectual innovation, patent is often used to analyze technological trends, cooperation between different patentees, and the status of the investigated company in the industry [32].

Based on the above literature review, it is found that scholars have touched upon the value of cross-border innovation diffusion, the behaviors of innovation, collaboration and dissemination within innovation networks are still worth further exploration In particular from a long-term perspective of regional policy, the changes in inter-city collaborative patent networks require further study. Currently, most research focus on multiple cities within one country or innovation networks among multiple countries under the same system. The Greater Bay Area, as a region with a unique institutional framework, offers a distinct research perspective [33,34]. The changes and differences among collaborative subjects within the innovation networks still hold potential for further research. In the Greater Bay Area, the different characteristics in two different periods, before and after the national policy (*the Outline Development Plan for the Guangdong-Hong Kong-Macao Greater Bay Area*) launched in 2015, were rarely explored. While patent data effectively captures technology commercialization outcomes, the complexity of innovation processes necessitates multidimensional analysis incorporating research publications and R&D investment metrics. Given this study’s focus on policy-driven technology transfer pathways, patent data demonstrates stronger policy relevance and traceability. Consequently, our investigation prioritizes collaborative patent data as the analytical foundation. The article will examine characteristics of innovation cooperation network in the Greater Bay Area in a long term from 2010 to 2022, particularly the differences of innovation cooperation network before and after 2015, processing in depth research in this field.

This paper aims to investigate the models and features of the scientific and technological innovation cooperation network in the Greater Bay Area. We choose 2015 as the year of policy implementation and examine the different characteristics of collaboration policy on cross-border knowledge flow before and after 2015 by exploring the co-patents of this region via the social network analysis. The rest of the article proceeds as follows: The Data and methods section details the methods and metrics used in the article; Next, the article introduces the evolutionary characteristics of innovation cooperation in science and technology within the Greater Bay Area; Finally, the Conclusion and discussion section summarizes the findings of the analysis in the innovation cooperation network of the Greater Bay Area, and also provides some recommendations.

## Data and methods

### Research area and data source

The Greater Bay Area comprises nine cities in Guangdong Province: Guangzhou, Shenzhen, Zhuhai, Foshan, Huizhou, Dongguan, Zhongshan, Jiangmen, and Zhaoqing, alongside Hong Kong and Macao. The Greater Bay Area, a unique region, operates under “one country, two systems”, “three customs territories”, and “three legal systems”. It is one of the most thriving and accessible regions in China, with significant strategic importance for the overall national advancement. In 2015, the central government officially recognised the Greater Bay Area as a national level development region. This study concentrates on the 11 cities within the Greater Bay Area and divides patent cooperation in the Greater Bay Area into two stages from 2010 to 2022: the first stage from 2010 to 2015 and the second stage from 2016 to 2022. Given the study’s focus on policy-driven technology transfer, collaborative patent data offer strong relevance and traceability in measuring cross-border innovation. Patents, particularly those with multiple applicants from different cities, reflect not only technological output but also institutional cooperation. Compared to publication or R&D investment data, co-patents more directly capture the spatial and institutional linkages necessary for social network analysis. Furthermore, the directional and relational nature of co-patent data allows us to map actual innovation collaboration patterns within the Greater Bay Area. But in a way, the research takes a limited definition of patent collaboration, specifically co-signed or jointly filed collaborative patents [35], and analyses cooperative invention patent data between cities in the Greater Bay Area. The analysis focuses on patent applicants who have jointly applied for patents and have at least two addresses belonging to Guangdong, Hong Kong, and Macao.

The patent cooperation data utilised in this study was sourced from the patent search and analysis website of the China National Intellectual Property Administration (https://pss-system.cponline.cnipa.gov.cn/conventionalSearch). To ensure data quality and mitigate bias, we adopted several filtering criteria. First, the data set is limited to patents disclosed between January 1, 2010 and December 31, 2022, and was used in establishing the original database. Second, the patent cooperation was screened based on the “applicants” in the patent database. If at least one applicant comes from the nine cities of Guangdong Province and at least one from Hong Kong or/and Macao, then scientific and technological innovation cooperation is deemed to have been established. If the applicants include units outside the Greater Bay Area, the patent cooperation information of the Greater Bay Area is also covered. Additionally, the patent screening process implemented rigorous validity verification: only patents maintaining legal status throughout the research period were retained, while expired patents were systematically excluded. Furthermore, the selected patent data were ensured to comprehensively cover strategic emerging industries emphasized in policy blueprints, particularly advanced manufacturing, new materials, and information equipment. This reflects structural disparities rather than data omission.The final curated dataset comprises 531 valid patent records. More data can be seen in the supplementary materials. Geospatial mapping in this study utilized the official Guangdong-Hong Kong-Macao Greater Bay Area base map (GS(2019)4342). It obtained from the Standard Map Service portal administered by the National Geomatics Center of China (NGCC, http://bzdt.ch.mnr.gov.cn/). This cartographic resource is publicly accessible for download and use.

### Methods and indicators

Social network analysis, as a developing research field, focuses on measuring and analyzing relational structures, which has been widely applied for empirical research since the early 20th century [36,37]. It employs a set of conventional research indicators with a unique conceptual system and measurement tools to scrutinize the structural and relational properties of social units (individuals, groups, or elements) by a series of nodes and node connections [38]. In the context of cross-border innovation, social network analysis provides a powerful conceptual and methodological framework to explore how innovation activities are structured, coordinated, and diffused across administrative boundaries. SNA treats cities as nodes and co-patenting ties as edges, allowing us to map and measure the structural features of the innovation system. Key network indicators—such as degree centrality, betweenness centrality, density, clustering coefficient, and core-periphery structure—reflect different aspects of the regional innovation dynamics. These indicators capture how innovation capacity is distributed, how efficiently knowledge flows across regions, and how institutional relationships evolve over time. By analyzing positional shifts in the network, we can trace how policy initiatives reshape the roles of cities and institutions in cross-border collaboration. In this paper, nodes in the network are cities for scientific and technological innovation cooperation. There are 11 nodes, including nine cities in Guangdong province and two special administrative regions. Co-patents among 11 cities of the Greater Bay Area are used as edges to construct an undirected network for scientific and technological innovation. The weight of the edge indicates the number of cooperative patents. As it aims to explore the evolution of scientific and technological innovation cooperation in the Greater Bay Area, this paper will analyze two different periods to examine the differences. Moreover, this paper also analyzes the characteristics of scientific and technological innovation cooperation network in the Greater Bay Area at two levels: overall level and node level. The paper conducts visualization and analysis through Ucinet. As this study uses publicly available patent data and does not involve human subjects, no ethical approval was required. Table 1 presents the specific network evaluation indexes and calculation methods used.

**Table 1:** Evaluation indicators of patent innovation cooperation network in the Guangdong- Hong Kong-Macao Greater Bay Area.

Network	Indicators	Calculation formula	Indicator Description	Definition
	Number of nodes	N	N: The subject’s corresponding vertex	Subjects involved in cooperation
	Number of sides	L	L: Connection between subjects	Number of cooperative node connections
Whole level	Network Density	$\begin{array}{c} d=\frac{2l}{n(n-1)}\sum_{i\gg j}d_{ij} \end{array}$	N: number of nodes L: number of sides	The compactness of node cooperation in a network
	Average path length	$\begin{array}{c} L=\frac{1}{n(n-1)}\sum_{i\neq j\in v}d_{ij} \end{array}$	$d_{ij}$ refers to the shortest distance between nodes i and j	The speed of information transfer in the network
	Clustering coefficient	$\begin{array}{c} CC=\frac{1}{n}\sum_{i=1}^{n}\left[\frac{{2e}_{i}}{k_{i}}\left(k_{i-1}\right)\right] \end{array}$	$k_{i}$ refers to the core degree of node i, $e_{i}$ refers to the number of edges between neighbors	Relevance of node in the network
	core-periphery	$\begin{array}{c} \rho =\sum_{i,j}\alpha_{ij}\delta_{ij} \end{array}$	$\alpha_{ij}$ indicates whether there is a cooperative relationship, and $\alpha_{ij}$ = 1 if there is a relationship between i and j, and 0 otherwise.	Core or edge position of the node in the network
	Degree centrality	$\begin{array}{c} C_{AD}\frac{\sum_{i=1}^{n}\left(C_{{AD}_{\max}}-C_{{AD}_{i}})\right]}{max[\sum_{i=1}^{n}\left(C_{{AD}_{\max}}-C_{{AD}_{i}})\right]} \end{array}$	$C_{AD}$ is the absolute centrality of point i, and $C_{{AD}_{\max}}$ is the absolute centrality of the points in the graph.	The degree of centrality of a node among its neighboring nodes with which it cooperates
Node level	Betweenness centrality	$C_{B}=\frac{\sum_{i=1}^{n}\left(C_{{RB}_{\max}}-C_{{RB}_{i}}\right)}{n-1}$	$C_{{RB}_{i}}$ is the relative intermediate centrality of the point, and $C_{{RB}_{\max}}$ is the maximum value that node i may reach.	Degree of control of resources by nodes
	Closeness centrality	$C_{X}=\frac{N}{\sum_{y}d(y,x)}$	d(y,x) is the average of the shortest distance between nodes y and x, representing the total number of nodes.	The central position of the node at the geometric level of the network

## Evolutionary characteristics of the scientific and technological innovation cooperation in the Greater Bay area

### Visualization of scientific and technological innovation network in the Greater Bay Area

**Fig 1:**
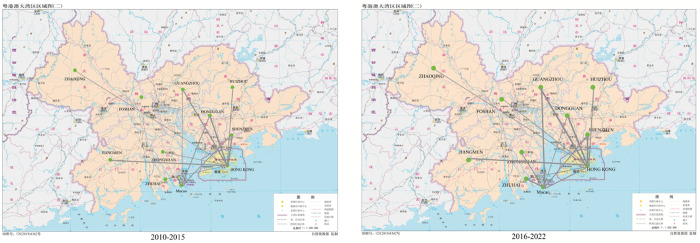
Scientific and Technological Innovation Network of the Guangdong-Hong Kong- Macao Greater Bay Area (2010-2022) (Fig 1 obtained from the Standard Map Service portal administered by the National Geomatics Center of China (NGCC, http://bzdt.ch.mnr.gov.cn/)).

The innovation cooperation network of science and technology in the Greater Bay Area is modelled as an undirected network in this paper. Processed data is employed to create a visualization map of the patent cooperation innovation network in the Greater Bay Area for the two phases spanning 2010–2015 and 2016–2022 (Fig 1). The creation of the cooperation network map enables the visualisation of each city’s position in the scientific and technological innovation cooperation network, as well as the intensity of collaboration, and the radiating effect the core city has. This provides a solid foundation for network analysis.

Fig 1 presents the visualized network of intercity innovation cooperation based on co-patent relationships. It depicts 11 nodes, which represent Hong Kong, Macao, and nine cities in Guangdong Province: Guangzhou, Shenzhen, Zhuhai, Foshan, Huizhou, Dongguan, Zhongshan, Jiangmen, and Zhaoqing. The connecting line between nodes indicates patents jointly applied by the cities, with the thickness indicating the level of cooperation between actors. The greater the thickness of the line between two nodes, the more patents the two cities have collaborated on. Additionally, according the result and natural breaks (Jenks) method, scientific and technological innovation cooperation intensity is graded based on quartiles, classified as low cooperation intensity (0–3), relatively low cooperation intensity (4–9), medium cooperation intensity (10–26), and high cooperation intensity(27,179),with higher levels of cooperation between cities resulting in higher grades. The characteristics of the evolution of scientific and technological innovation cooperation in the Greater Bay Area can be observed from 2010 to 2022. From 2010–2015, the intensity of cooperation among Guangzhou, Hong Kong, and Shenzhen within the science and technology innovation cooperation network of the Greater Bay Area was characterized as high. The network structure was relatively simple, centered around the connections between “Guangzhou-Hong Kong” and “Shenzhen-Hong Kong”. However, isolated nodes still existed, as Foshan had not yet participated in the cooperation network. Additionally, no cooperative relationships had been established among Hong Kong, Huizhou, Zhuhai, Foshan, Zhongshan, Zhaoqing, Jiangmen, and Macao, resulting in a loosely connected network. By 2016–2021, the cooperation network became more compact, with no remaining isolated nodes. The intensity of regional cooperation increased, and most cities were in a state of low to medium cooperation intensity. Gradually, a science and technology cooperation network has formed, and centered around the connections between “Shenzhen-Hong Kong”, “Guangzhou-Hong Kong” and “Guangzhou- Macao”. Although the cooperation network became increasingly close and the intensity of cooperation grew, Macao still lacked cooperative links with Foshan, Huizhou, Zhaoqing, and Jiangmen. Overall, despite the proposal for regional cooperation by the governments of Guangdong, Hong Kong and Macao in 2010, Macao still has room for improvement in implementing cross-regional cooperation within the Greater Bay Area.

The network initially had a loose structure that became increasingly close over time. Currently, the network is more open and centered around Hong Kong-Shenzhen-Guangzhou. However, the scientific and technological innovation cooperation within the Greater Bay Area displays an irregular pattern, and the cooperative network is still in the developmental stage.

**Fig 2:**
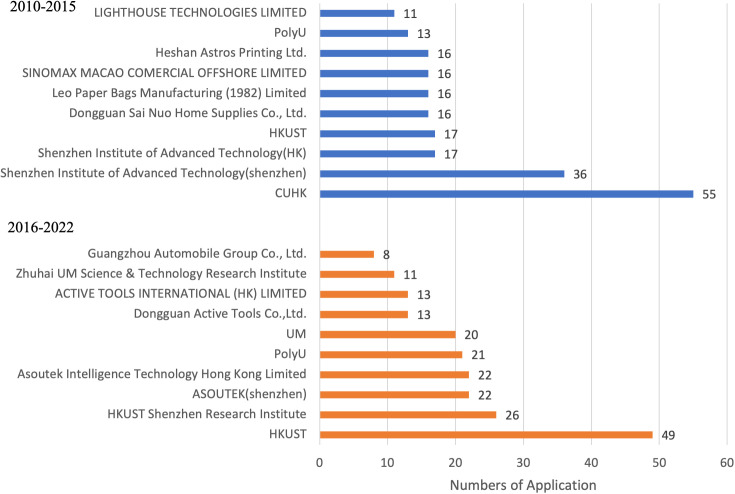
Core patent applicants of the Guangdong-Hong Kong-Macao Greater Bay Area (2010-2022).

**Fig 3:**
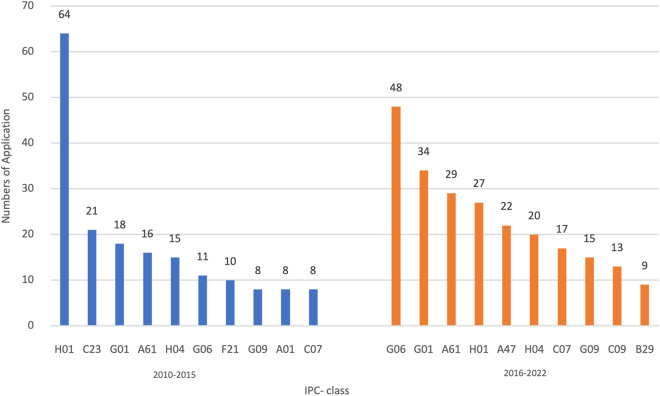
Technological distribution of cooperated patents in the Guangdong-Hong Kong-Macao Greater Bay Area (2010-2022).

Fig 2 further illustrates how the composition of major patent applicants evolved geographically. It depicts the shift in primary candidates over two phases. From 2010 to 2015, collaborative patents were primarily dominated by Hong Kong universities, research institutions partnering with Shenzhen, and enterprises, while Macao and mainland enterprises exhibited limited participation. However, in the subsequent phase, the geographical distribution of applicants became more evenly distributed. Hong Kong’s diverse innovation entities continued to be active, while universities and collaborative research institutes in Macao increased their involvement. A significant proportion of patents in both regions resulted from university collaborations. Fig 3 provides a comprehensive breakdown of core co-patents by their technological distribution. These patents have been classified based on the International Patent Classification List (IPC). Fig 3 provides insight into the technological fields of collaborative innovation, using IPC classification to identify dominant areas of patenting activity. It displays the top 10 core patents analyzed in the broad category indicators of IPC Stages One and Two. These include A. essentials of human life, B. operations and transportation, C. chemistry and metallurgy, D. textile and paper, E. fixed buildings, F. mechanical engineering, illumination, heating, weaponry, and explosives, G. physics, and H. electricity. In the initial period, H01 (fundamental electrical components) witnessed a substantial growth in usage, whereas the other primary categories had more modest growth. Taking the major categories of the first stage as a reference, there was a rise in the total number of applications during the second stage, except for a decrease in H01. The category with the most significant growth was G06 (instrumental calculation). The innovative restructuring has been steadily influenced by the impact of enhancing people’s lives and promoting high-quality development of science and technology. This can be observed through the rising projections in sectors like G01 (instrument measurement and testing) and A61 (medicine/veterinary medicine and sanitation), which entail objective quantification.

### Overall level characteristics

The assessment of a network’s comprehensive level concerns the composition and relationships of all members within a group. Its structural characteristics are evaluated through various indicators including network size, network density, average path length, clustering coefficient, core-periphery structure, and others.

#### Network size and density.

Table 2 provides an overview of the essential properties of the scientific and technological innovation network in the Guangdong-Hong Kong-Macao region. The data shows that, in the first stage, Foshan acted as an “isolated point”. However, all 11 cities from Guangdong, Hong Kong and Macao actively participated in the scientific and technological innovation cooperation network during the following phase. Additionally, the number of cooperative relationships increased from 227 to 357 at the second stage, indicating a growing scope for scientific and technological innovation collaboration within the Greater Bay Area. The connections between the main parties illustrate a developing closeness. Collaboration among nodes appears to rise as the network density and compactness increase. The density of networks in scientific and technological innovation collaboration among Guangdong, Hong Kong and Macao has shown an upward trajectory from 0.2 at the first phase to 0.2545 at the second phase from 2010 to 2022. The rise in scientific and technological collaboration between these regions indicates an increase in both frequency and openness. However, the network’s density is relatively low overall, and the structure exhibits decentralization. This could indicate two problems: first, it appears that the network may not provide sufficient support and influence to all 11 participating cities; second, there may be a lack of closeness between the cities which contributes to ineffective cooperation, low levels of cooperation, and limited circulation of knowledge and innovation resources. From the perspective of the density of the patent cooperation and innovation network in the Greater Bay Area, the network density is relatively low. Although this situation does not necessarily indicate that the relationships among all cities are loose, in the long run, the fragmentation of the patent cooperation and innovation network may have a negative impact on the coordinated development of scientific and technological innovation in the Greater Bay Area.

**Table 2:** Overall basic characteristics of patent innovation cooperation network in the Guangdong-Hong Kong-Macao Greater Bay Area.

Index	2010-2015	2016-2022
Number of nodes	10	11
Number of lines	227	357
Network density	0.2	0.2545
Average distance	1.867	1.818
Clustering coefficients	0	0.730

#### Average path length and clustering coefficient analysis.

Jeffrey Travers and Stanley Milgram utilized average path length and clustering coefficient to illustrate the “small world” theory through conducting experiments on small groups. The “small world” theory posits that any two persons in the world can be connected through six persons, thereby making the world a small world [39]. A shorter average distance length facilitates convenient connection between nodes, and a larger clustering coefficient results in closer cooperation between nodes. When the average path length of a network is small but its clustering coefficient is large, it indicates that the network possesses small-world characteristics. The criterion for determining a small-world network is that the average path length of the network is less than 10 clustering coefficients but greater than 0.1, with possible adjustments for networks with many nodes [40–42]. From Table 2, it is evident that the average path length of the patent innovation collaboration network in the Greater Bay Area has decreased from 1.867 to 1.818 and the clustering coefficient has increased from 0 to 0.730. Based on the characteristics of the small-world effect, it suggests that the “bridging” effect of the cooperative network is boosted, facilitating cooperation between cities and increasing the likelihood that cities will seek to strengthen cooperation and promote mutual understanding. Opting to enhance collaboration and gain deeper insight into each other’s innovation status in science and technology can boost the transfer of resources and knowledge in the network, enhancing communication and dialogue among cities, and ultimately elevating their respective innovation capabilities in science and technology.

#### Core-periphery structure.

The core-periphery model’s appropriateness for measuring the innovation cooperation network within the Greater Bay Area is gauged by using UCINET software. If there is cooperation between a city of Guangdong Province and Hong Kong and/or Macao, it is recorded as “1”, in contrast, if there is no cooperation, it is recorded as “0”. The UCINET software produces two scores --- 0.979 and 1, respectively for one period, which efficiently portrays the core-periphery structure of the innovation cooperation network in the Greater Bay Area. In fact, 11 cities in the Greater Bay Area also get the coreness in the innovation cooperation network in two periods, which can be classified into three kinds: core (coreness > 0.1), sub-core (0.01 < coreness < 0.1), periphery (coreness < 0.01) [43]. According to the division of coreness (Table 3), from 2010 to 2015, Hong Kong was located in the core area, while Shenzhen and Dongguan were in the sub-core area, with the remaining cities being in the peripheral zone, indicating that the science and technology innovation cooperation network had not yet formed. By 2016–2022, the core area expanded to include Hong Kong, Guangzhou, and Shenzhen, while Dongguan remained in the sub-core area, and the other cities were classified as peripheral zones. However, during this period, the coreness of all cities increased to some extent. Overall, within the science and technology innovation cooperation network, Hong Kong, Shenzhen, and Guangzhou gradually occupied the core positions. To a certain extent, these three cities were not only engaged in comprehensive cooperation but also enhanced the depth and intensity of participation in the cooperation network by surrounding areas, although their radiating influence was not yet pronounced. Additionally, in terms of coreness, Hong Kong maintained a dominant position in the core area. From 2010 to 2022, its coreness generally exhibited an upward trend, and the core-periphery structure remained evident.

**Table 3:** Coreness of patent innovation cooperation network in the Guangdong-Hong Kong-Macao Greater Bay Area.

CITY	2010-2015	2016-2022
Honhkong	0.999	0.999
Macau	0.002	0.008
Guangzhou	0.009	0.014
Shenzhen	0.041	0.048
Foshan	0.000	0.001
Huizhou	0.003	0.009
Zhuhai	0.000	0.001
Dongguan	0.012	0.013
Zhongshan	0.001	0.004
Zhaoqing	0.000	0.001
Jiangmen	0.004	0.005

### Node level characteristics

This section analyses crucial nodes within the cooperation network from a micro perspective. The aim is to reflect each city’s status and position in the science and technology innovation network, mainly through centrality indicators. Table 4 displays degree centrality, betweenness centrality, and closeness centrality indicators of Guangdong, Hong Kong, and Macao. Table 4 includes the node characteristics of the leading 10 patent applicants for each period to examine the node characteristics of the scientific and technological innovation cooperation network in the Greater Bay Area.

**Table 4:** Node level characteristics of patent innovation cooperation network in the Guangdong-Hong Kong-Macao Greater Bay Area.

Indicators	Phase	Indicators explanation	Hong Kong	Macao	Guang zhou	Shen zhen	Fo shan	Hui zhou	Zhu hai	Dong guan	Zhong shan	Zhao qing	Jiang men
**Degree centrality**	Phase I	Degree	217	10	29	132	0	8	1	43	2	1	11
	Phase II	Degree	308	57	76	183	4	7	25	38	4	2	10
**Betweenness centrality**	Phase I	Betweenness	31.5	1.5	2	2	0	0	0	2	0	0	0
	Phase II	Betweenness	33	12	0	0	0	0	0	0	0	0	0
**Closeness centrality**	Phase I	Farness	21	31	27	27	0	29	29	27	29	29	29
	Phase II	Farness	11	14	18	18	20	20	18	18·	13	20	20

Table 4 shows the centrality characteristics of each node in the patent innovation cooperation network of the Greater Bay Area. In terms of degree centrality, in the phase I, the degree centrality of Hong Kong, Shenzhen, Guangzhou and Dongguan is high, indicative of their core position in the network, and enabled by independent innovation, while the degree centrality of other cities is relatively low and denotes their reliance on scientific and technological innovation of the core cities. This situation has been improved at the second stage. In addition to the four cities prominent at the first stage, Macao and Zhuhai gradually move closer to the core region. On the betweenness centrality, Hong Kong, Macao, Shenzhen, Guangzhou and Dongguan act as bridges in the invention cooperation network at the first stage, while only Hong Kong and Macao play the critical intermediary role at the second stage. The primary cause may depend upon that this paper focuses on cross-border patent cooperation rather than the inter-cities cooperation within Guangdong Province [44,45]. Only co-patents have at least one applicant from Hong Kong and/ or Macao can be covered in the research. Not many cross-border co-patents have been observed. The less developed cities lacked the innovation cooperation with Hong Kong and Macao, thus their values of betweenness centrality are 0. At the second stage, as more and more cities have opportunities to establish the direct cooperation relation with Hong Kong and/ or Macao under the Greater Bay Area scheme, the bridge role of Shenzhen, Guangzhou and Dongguan in the invention cooperation network in the Greater Bay Area weakens. In terms of closeness centrality, the overall value of cities at the second stage decreases compared with those at the first stage, which may be due to that the number of cities that need to go through multiple nodes to achieve scientific and technological innovation decreases. Foshan is the most obvious case. Compared with the first stage, Foshan earns one central position in the network at the second stage.

In sum, compared to the first stage, cities with weaker control over resources and information gradually show their own level of science and innovation development at the second stage. In addition, in the second stage, Hong Kong and Shenzhen persist in their radiating roles. However, during this period, Guangzhou and Macao’s social relations undergo significant strengthening, particularly Macao’s emerging innovative autonomy. This, to a large extent, permits them to influence other nodes, shortening the path of cooperation with other cities and resulting in a new core area.

The Greater Bay Area Development Plan in 2015 endorses the collaboration of businesses, universities, and research institutions in Guangdong, Hong Kong, and Macao to establish advanced collaborative innovation platforms that facilitate the conversion of scientific and technological achievements. The plan also enables qualifying universities and research institutions in Hong Kong and Macao to apply for mainland scientific and technological research projects and collaborate on major scientific research programmes, which has significantly elevated their scientific achievement. The impetus for engaging in innovation activities in Guangdong, Hong Kong, and Macao, along with universities’ inclination towards innovation components in collaborative innovation, is robust. This provides a solid basis for establishing a center of scientific and technological innovation.

Table 5 reveals a significant rise in the role of universities and research institutes as key intermediaries in the innovation cooperation network of the Greater Bay Area. Institutions such as the Hong Kong University of Science and Technology (HKUST), the Hong Kong Polytechnic University (PolyU), the Macau University of Science and Technology (UMST), Sun Yat-sen University (SYSU), and others demonstrate exceptionally high betweenness centrality values in the second period (2016–2022), indicating their strategic position in bridging otherwise disconnected nodes within the network. Betweenness centrality reflects a node’s capacity to act as a conduit for knowledge flows between actors that are not directly connected. The high scores of these universities suggest that they occupy critical “broker” positions, facilitating cross-regional knowledge exchange, accelerating the diffusion of innovation, and enabling access to resources across institutional boundaries. This is particularly notable in a cross-border context like the Greater Bay Area, where legal, administrative, and systemic differences present barriers to direct collaboration. The increasing centrality of universities stems from both policy incentives and intrinsic institutional advantages. On one hand, since the launch of the Greater Bay Area Outline Plan, central government policies have explicitly supported joint R&D initiatives and cross-border academic cooperation, granting Hong Kong and Macao universities access to mainland research funding and collaborative platforms. On the other hand, universities inherently possess strong research capacity, multidisciplinary teams, and flexible institutional structures, allowing them to adapt quickly and act as trusted, neutral intermediaries between firms, governments, and other knowledge actors. Furthermore, the growing presence of institutions from both Hong Kong (e.g., HKUST, CUHK) and Macao (e.g., UMST, UM) in top intermediary positions signals a gradual institutional rebalancing. This not only reflects improved innovation autonomy in Hong Kong and Macao, but also suggests a more inclusive innovation ecosystem where smaller or less industrially dominant cities can still play critical roles through their academic institutions.

**Table 5:** Betweenness centrality of patent innovation cooperation network node of the Guangdong-Hong Kong-Macao Greater Bay Area.

	Patent Application	Betweenness Centrality
2010-2015	UMST	6
2016-2022	HKUST	1896
	PolyU	1383
	UMST	1250.667
	SYSU	603
	Huawei Technologies Co.Ltd.	595
	SCUT	595
	SUST	564
	UM	500
	CUHK	478

## Discussion and conclusion

Currently, the cross-border innovation system in the Greater Bay Area is relatively remains relatively underdeveloped. Although a network of scientific and technological innovation collaboration has been established, there is a noticeable tendency for innovation entities to partner with those that exhibit higher levels of economic development, administrative authority, and network status.

From 2010 to 2022, although the density of intercity networks is low in both phases and communication and cooperation between cities are relatively small and loosely structured, the overall network scale has increased, and density has been on the rise. Simultaneously, the network structure demonstrated several positive developments, including shorter path lengths, higher clustering coefficients, and an increasing number of connections. These features suggest a rise in intensive cooperation and exchanges among cities, reflecting a shift toward more integrated and efficient collaboration. As a result, the network has achieved greater cohesion, with a core structure gradually emerging around Hong Kong, Shenzhen, and Guangzhou. These three cities have become central hubs, driving innovation and fostering stronger regional integration. Their dominant positions within the network highlight their pivotal roles in shaping the GBA’s innovation landscape. However, the network also reveals significant imbalances, particularly concerning Macao. Despite the overall progress, Macao’s participation in the patent innovation cooperation network remains limited. At present, Macao lacks cooperative relationships with four of the nine cities in Guangdong Province—Foshan, Huizhou, Zhaoqing, and Jiangmen. This fragmented and uneven collaboration landscape underscores Macao’s relative isolation within the regional innovation ecosystem. Several factors may contribute to this disparity, including Macao’s small land area, limited population, and homogenous industrial structure, which is heavily reliant on tourism and gaming.

One interesting observation is that, according to the node level characteristics of patent innovation cooperation network, the current cross-border cooperation in scientific and technological innovation in the Greater Bay Area is mostly concentrated in point-to-point cooperation between cities in Guangdong Province and Hong Kong or Macao rather through Guangzhou or Shenzhen. Nevertheless, intermediary institutions can still act as bridges to enhance the spatial structure of scientific and technological innovation cooperation in the Greater Bay Area. This can also facilitate the integration of periphery cities in the same region. The node status in the network has an asymmetrical significance and influence. With the passage of time, Guangzhou and Macao have ascended in rank within the Guangdong-Hong Kong-Macao patent innovation cooperation network, occupying a more central position. Hong Kong, Macao, Guangzhou and Shenzhen wield greater control over resources for scientific and technological innovation, exhibit stronger willingness and capability for cooperation in this field, and exert more assertive influence. Furthermore, central policy changes have made universities and companies important contributors to the joint patent application process. The bolstering of inter-city connections, through integration into collaborative networks and establishing links with other nodes via intermediaries, significantly influences the scientific and technological innovation collaboration. The nodes’ autonomy and centrality are reflected in their proximity to the center, with universities and companies of Hong Kong, Shenzhen, and Macao exhibiting superior scientific and technological innovation independence. Due to differences in geography, institutional environment, and infrastructure, there is an imbalance in the power and resource control of cities within the network. This imbalance hinders both the efficiency and stability of the network. To address this issue, cities must position themselves clearly, utilize their strengths in resources, and enhance cooperation in scientific and technological innovation to promote complementarity and strengthen the network’s cohesion.

Based on the aforementioned conclusions, it is clear that the scientific and technological innovation cooperation in the Greater Bay Area has both advantageous and disadvantageous aspects. Since the construction of the Greater Bay Area, there has been a significant expansion in the scale of scientific and technological innovation cooperation and an increase in the cohesion of cross-border collaboration. The 13th Five-Year Plan in 2016 proposed the construction of a platform for the cross-regional cooperation in scientific and technological innovation in the Greater Bay Area, which represents an unprecedented historical opportunity for deepening and expanding cooperation in this field. The Greater Bay Area Development Plan, released in 2019, aims to establish the Greater Bay Area as a prominent regional centre for scientific and technological innovation. This plan has been successful in facilitating the flow of scientific and technological innovations and providing the necessary infrastructure for a collaborative innovation network. In contrast, the cross- regional cooperation in the scientific and technological innovation level seems to remain inactive. Additionally, the node indicators demonstrate that the dissimilarities in geographical location, institutional environment and infrastructure have produced substantial asymmetry in the power and resource management capabilities of various cities within the network, which have thwarted the effectiveness and stability of the network structure. The findings from the social network analysis also provide a structural lens to understand how the innovation system has evolved under policy influence. For instance, the increased network density and clustering coefficient reflect enhanced connectivity and more efficient knowledge sharing, while the gradual formation of a core-periphery structure reveals centralization in key cities such as Hong Kong, Shenzhen, and Guangzhou. Changes in node-level centrality suggest not only shifts in innovation capability but also realignments in institutional collaboration patterns. These structural dynamics reveal how the cross-border innovation system is becoming more integrated yet still fragmented, especially with regard to the peripheral cities.

In terms of essential patent applicant cooperation, the first stage primarily involves standardized collaboration among universities, research institutes, and companies with dual locations operating in an operation mode of “front store and back factory”. Moving into the second stage, university-enterprise cooperation has increased proportionally, and numerous enterprises aim to join the innovation network through cooperative efforts with one another. According to the centrality index of patent applicants, universities act as strong intermediaries in innovation cooperation. The top-ranked institutions are mainly well-known universities with greater openness and inclusivity in the scientific and technological innovation cooperation, a stronger cooperation ethos and willingness to collaborate, and are pivotal in the entire network of scientific and technological innovation cooperation. In terms of technical fields of collaboration among core applicants, a progression has been observed between the first and second stages, where companies specialising in “inspection”, “intelligence” and “electronics” within bio-detection and high-tech industries have emerged in the second stage. In the second phase, they are steadily developing, particularly in the field of science and technology, and actively pursuing partnerships with renowned universities to enhance industrial growth. Living labs, understood as innovation projects based on open and user-centric innovation methodologies, can form collaboration networks to support small firms and other actors to engage in cross-border collaboration and to accelerate the development and acceptance of innovations [46]. Governments in the Greater Bay Area may take actions to promote living labs for the cross-border collaboration.

Hence, it is imperative for the authorities to consistently revamp the mechanism. There are four approaches, one is that, for less developed cities, enhancing patent cooperation in such cities can be achieved by establishing branches of joint research institutes or laboratories in these areas and integrating them into the R&D projects of core cities. The second is to comprehensively consider regional collaboration mechanisms to formulate policies such as cross-city R&D tax incentives, reductions in licensing fees, and the establishment of inter-regional patent sharing pools. Thirdly, facilitating the influx of invention resources and constituents in the Greater Bay Area, create a forum for talent interchange, and promptly implement incentive schemes to augment innovative collaboration amidst academia, corporations, and research establishments. Furthermore, universities and research institutions ought to take into account the conversion rate of collaborative patents to enhance the caliber of their innovation and research and development. Different systems present both barriers and opportunities. Patent research and development should aim to seek external incentives to expand the market.

## Supporting information

S1 File Orginal data. (XLSX)

## References

[1] Li L, Man L, Yang S. Institutional differences and institutional innovation: reforms in the Greater Bay Area under the multiple institutional interaction. Rev Public Admin. 2020;2:23–39.

[2] Wang J, Chandra K, Du C, Ding W, Wu X. Assessing the potential of cross-border regional innovation systems: a case study of the Hong Kong -Shenzhen region. Technol Soc. 2021;65:101557. doi: 10.1016/j.techsoc.2021.101557

[3] Koschatzky K. A river is a river-cross-border networking between Baden and Alsace. Eur Plann Stud. 2000;8(4):429–49. doi: 10.1080/713666422

[4] Platonov V, Bergman J-P. Cross-border cooperative network in the perspective of innovation dynamics. Int J Knowl-Based Organ. 2011;1(1):1–19. doi: 10.4018/ijkbo.2011010101

[5] Verspagen B. Uneven growth between independent economies: An evolutionary view on technology gaps, trade and growth. Avebury UK: Aldershot; 1993.

[6] Boschma R, Frenken K. The spatial evolution of innovation networks: A proximity perspective. The handbook of evolutionary economic geography. Edward Elgar Publishing; 2010.

[7] Cappellano F, Makkonen T. Cross-border regional innovation ecosystems: the role of non-profit organizations in cross-border cooperation at the US-Mexico border. GeoJournal. 2019;85(6):1515–28. doi: 10.1007/s10708-019-10038-w

[8] Lundquist KJ, Trippl M. Towards cross-border innovation spaces: A theoretical analysis and empirical comparison of the Öresund region and the Centrope area. 2019.

[9] Weidenfeld A. Tourism and cross border regional innovation systems. Ann Tour Res. 2013;42:191–213. doi: 10.1016/j.annals.2013.01.003

[10] OECD. Regions and innovation: Collaborating across borders, OECD Reviews of Regional Innovation. OECD Publishing; 2013.

[11] Sohn C. Modelling cross-border integration: the role of borders as a resource. Geopolitics. 2014;19(3):587–608. doi: 10.1080/14650045.2014.913029

[12] Li Y. Implications of government-industry-university-research collaborative innovation in the San Francisco Bay Area for the Guangdong-Hong Kong-Macao Greater Bay Area. J South China Univ Technol (Social Science Edition). 2020;22(1):1–11.

[13] Cappellano F, Sohn C, Makkonen T, Kaisto V. Bringing borders back into cross-border regional innovation systems: functions and dynamics. Environ Plann A: Econ Space. 2022;54(5):1005–21. doi: 10.1177/0308518x221073987

[14] Zhang Y, Gao C, Wang J. Financing constraints and innovation performance: The moderating role of the network location of cross-border innovation cooperation among Internet enterprises. Eur J Innov Manag. 2022.

[15] Yang Z, Shen C, Lam FI. Scientific and technological innovation and cooperation in the Greater Bay Area of China: a case study of university patent applications and transformation. Sustainability. 2024;16(2):571. doi: 10.3390/su16020571

[16] Xinhua News Agency. The Outline Development Plan for the Guangdong-Hong Kong-Macao Greater Bay Area. 2019. Available from: https://www.gov.cn/zhengce/2019-02/18/content_5366593.htm#1

[17] Yu Q. Study on the Guangdong-Hong Kong-Macao Greater Bay Area. Modern Econ. 2019;10(03):586–99. doi: 10.4236/me.2019.103040

[18] Song C, Sun C, Xu J, Fan F. Establishing coordinated development index of urbanization based on multi-source data: a case study of Guangdong-Hong Kong-Macao Greater Bay Area, China. Ecol Indic. 2022;140:109030. doi: 10.1016/j.ecolind.2022.109030

[19] Li C, Ng MK, Tang Y, Fung T. From a ‘World Factory’ to China’s Bay Area: a review of the outline of the development plan for the Guangdong-Hong Kong-Macao Greater Bay Area. Plann Theor Pract. 2021;23(2):310–4. doi: 10.1080/14649357.2021.1958539

[20] Makkonen T, Williams AM, Mitze T, Weidenfeld A. Science and technology cooperation in cross-border regions: a proximity approach with evidence for Northern Europe. Eur Plann Stud. 2018;26(10):1961–79. doi: 10.1080/09654313.2018.1500528

[21] Tang C, Qiu P, Dou J. The impact of borders and distance on knowledge spillovers – evidence from cross-regional scientific and technological collaboration. Technol Soc. 2022;70:102014. doi: 10.1016/j.techsoc.2022.102014

[22] Ma H, Xu X. The effects of proximities on the evolving structure of intercity innovation networks in the Guangdong–Hong Kong–Macao Greater Bay Area: comparison between scientific and technology knowledge. Int J Urban Sci. 2022;27(3):390–413. doi: 10.1080/12265934.2022.2085154

[23] Yang W, Fan F, Wang X, Yu H. Knowledge innovation network externalities in the Guangdong–Hong Kong–Macao Greater Bay Area: borrowing size or agglomeration shadow? Technol Anal Strat Manag. 2021;34(9):1020–37. doi: 10.1080/09537325.2021.1940922

[24] Wang C, Ye Y, Huang Z. Synergistic development in the Guangdong-Hong Kong-Macao Greater Bay Area: Index measurement and systematic evaluation based on industry-innovation-infrastructure-institution perspectives. J Clean Prod. 2024;434:140093. doi: 10.1016/j.jclepro.2023.140093

[25] Zhao Y, Yongquan Y, Jian M, Lu A, Xuanhua X. Policy-induced cooperative knowledge network, university-industry collaboration and firm innovation: Evidence from the Greater Bay Area. Technol Forecast Soc Change. 2024;200:123143. doi: 10.1016/j.techfore.2023.123143

[26] Nagaoka S, Motohashi K, Goto A. Patent statistics as an innovation indicator. Handbook of the Economics of Innovation. Vol. 2. North-Holland: Elsevier. 2010. pp. 1083–127. doi: 10.1016/s0169-7218(10)02009-5

[27] Cai H, Feng Z, Zhou W, Chen J, Chen Z. Understanding the spatial polarization pattern of technology transfer network in the Guangdong–Hong Kong–Macao Greater Bay area. Growth and Change. 2022;54(1):4–25. doi: 10.1111/grow.12636

[28] Zare-Farashbandi F, Geraei E, Siamaki S. Study of co-authorship network of papers in the Journal of Research in Medical Sciences using social network analysis. J Res Med Sci. 2014;19(1):41–6. 24672564 PMC3963322

[29] Ter Wal ALJ, Boschma RA. Applying social network analysis in economic geography: framing some key analytic issues. Ann Reg Sci. 2008;43(3):739–56. doi: 10.1007/s00168-008-0258-3

[30] Sun H, Geng Y, Hu L, Shi L, Xu T. Measuring China’s new energy vehicle patents: a social network analysis approach. Energy. 2018;153:685–93. doi: 10.1016/j.energy.2018.04.077

[31] Blazsek S, Escribano A. Knowledge spillovers in US patents: a dynamic patent intensity model with secret common innovation factors. J Econom. 2010;159(1):14–32. doi: 10.1016/j.jeconom.2010.04.004

[32] Zhang L, Cao Z, Chen G, Wang Z. A study of China’s inter-city networks for innovation cooperation within software and service firms. Eur Geogr Econ. 2019;60(5):582–615. doi: 10.1080/15387216.2019.1695644

[33] Yang Z, Lin Z, Li L, Lam FI. The Guangdong-Hong Kong-Macao Greater Bay Area cooperation under ‘one country, two systems’: comparison with European Union and San Francisco Bay Area. Hong Kong J Soc Sci. 2022;58:1–29.

[34] Lin Z, Yang Z, Lam JFI, Li L. Guangdong–Hong Kong–Macao Cooperation: Historical Process and Driving Mechanisms. Soc Sci. 2024;13:297.

[35] Nagaoka S, Motohashi K, Goto A. Patent statistics as an innovation indicator. Handbook of the Economics of Innovation. Vol. 2. North-Holland: Elsevier. 2010. pp. 1083–127. doi: 10.1016/s0169-7218(10)02009-5

[36] Scott J. What is social network analysis? Bloomsbury Academic. 2012. pp. 114.

[37] Sosa S. Social network analysis. Encyclopedia of Animal Cognition and Behavior. Cham: Springer International Publishing; 2022. pp. 6527–44.

[38] Butts CT. Social network analysis: A methodological introduction. Asian J Soc Psych. 2008;11(1):13–41. doi: 10.1111/j.1467-839x.2007.00241.x

[39] Travers J, Milgram S. An experimental study of the small world problem. Sociometry. 1969;32(4):425. doi: 10.2307/2786545

[40] Valverde S, Cancho RF, Solé RV. Scale-free networks from optimal design. Europhys Lett. 2002;60(4):512–7. doi: 10.1209/epl/i2002-00248-2

[41] Humphries MD, Gurney K. Network “small-world-ness”: a quantitative method for determining canonical network equivalence. PLoS One. 2008;3(4):e0002051. doi: 10.1371/journal.pone.0002051 18446219 PMC2323569

[42] Latora V, Marchiori M. Efficient behavior of small-world networks. Phys Rev Lett. 2001;87(19):198701. doi: 10.1103/PhysRevLett.87.198701 11690461

[43] Ruan P, Wen G, Juan Y. Network structure and regional cooperation analysis of regional venture capital in China. Sci Technol Progr Policy. 2019;(14):8–17.

[44] Wen L i, Qiu Z h i. Research on the evolution of cooperative technological innovation in the Guangdong-Hong Kong-Macao Greater Bay Area based on patent measurement. Stat Res. 2019;36:74–86.

[45] Qiu Y, Ye Z, Pei L, Qing R. Research on the innovative network of Guangzhou, Dongguan and Shenzhen in the context of the Guangdong-Hong Kong-Macao Greater Bay Area development. City Plann Rev. 2021;(08).

[46] Schaffers H, Turkama P. Living Labs for Cross-Border Systemic Innovation. Technol Innov Manag Rev. 2012;2(9):25–30. doi: 10.22215/timreview/605

